# The Determining Effective Testing in Emergency Departments and Care Coordination on Treatment Outcomes (DETECT) for Hepatitis C (Hep C) Linkage-to-Care Trial: rationale and design of an emergency department-based randomized clinical trial of linkage-to-care strategies for hepatitis C

**DOI:** 10.1186/s13063-022-07018-w

**Published:** 2023-01-27

**Authors:** Sarah E. Rowan, Jason Haukoos, Kevin F. Kamis, Emily Hopkins, Stephanie Gravitz, Carolynn Lyle, Alia A. Al-Tayyib, Edward M. Gardner, James W. Galbraith, Yu-Hsiang Hsieh, Michael S. Lyons, Richard E. Rothman, Douglas A. E. White, Jake R. Morgan, Benjamin P. Linas, Allison L. Sabel, David L. Wyles, for The DETECT Hep C Trials Investigators, Amy Adler, Musheng Alishahi, Gideon D. Avornu, Alexis Becerra, Erika Becerra-Ashby, Meghan Bellamy, Samantha Bot, Alexander J. Boyle, Annetta M. Bracey, Michael Breyer, Claudia Camacho, Alicia Cupelo, Gaby Dashler, Pamela Doyle, Amy Eicher, Heather Gardner, Carrie Anne de Gruiter, Sophia Henry, David Higgins, Trevor Hill, Rachel Houk, Nyah Johnson, Alex Kile, Janet Liebl, Barbara Maliszewski, Kendall Maliszewski, Robert McGoey, Catherine McKenzie, Matthew S. Minturn, Deanna Myer, Kendra Neumann, Cole Ossian, Rebekah K. Peacock, Danielle Perez, Tannishtha Pramanick, Erin P. Ricketts, Benji Riggan, Sherry Riser, Genie Roosevelt, Mustapha Saheed, Sarah Schumacher, Bradley Shy, Scott Simpson, Matthew F. Toerper, Gil Trest, Madison Unsworth, Laura Waltrous, Brooke Watson

**Affiliations:** 1 Division of Infectious Diseases, Denver Health Medical Center and University of Colorado School of Medicine, Denver, CO USA; 2 Public Health Institute at Denver Health, Denver, CO USA; 3 Department of Emergency Medicine, Denver Health Medical Center and University of Colorado School of Medicine, 777 Bannock Street, Mail Code 0108, Denver, CO 80204 USA; 4 Department of Epidemiology, Colorado School of Public Health, Aurora, CO USA; 5 Colorado Social Emergency Medicine Collaborative, Denver, CO USA; 6 Department of Emergency Medicine, University of Mississippi Medical Center, Jackson, MS USA; 7 Department of Emergency Medicine, Johns Hopkins University, Baltimore, MD USA; 8 Department of Emergency Medicine, The Ohio State University Wexner Medical Center, Columbus, OH USA; 9 Department of Emergency Medicine, Highland Hospital, Alameda Health System, Oakland, CA USA; 10 Department of Health Law, Policy, and Management, Boston University School of Public Health, Boston, MA USA; 11 Center for Health Economics of Treatment Interventions for Substance Use Disorder, HCV, and HIV, Boston, MA USA; 12 Division of Infectious Diseases, Boston University School of Medicine, Boston, MA USA; 13 Department of Patient Safety and Quality, Denver Health, Denver, CO USA; 14 Department of Biostatistics, Colorado School of Public Health, Aurora, CO USA

**Keywords:** Hepatitis C, HCV, Linkage-to-care, Navigation, Clinical trial, Randomized trial, Emergency department, Comparative effectiveness, Methods, Implementation

## Abstract

**Background:**

Hepatitis C (HCV) poses a major public health problem in the USA. While early identification is a critical priority, subsequent linkage to a treatment specialist is a crucial step that bridges diagnosed patients to treatment, cure, and prevention of ongoing transmission. Emergency departments (EDs) serve as an important clinical setting for HCV screening, although optimal methods of linkage-to-care for HCV-diagnosed individuals remain unknown. In this article, we describe the rationale and design of The *D*etermining *E*ffective *T*esting in *E*mergency Departments and *C*are Coordination on *T*reatment Outcomes (DETECT) for *Hep*atitis *C* (Hep C) Linkage-to-Care Trial.

**Methods:**

The DETECT Hep C Linkage-to-Care Trial will be a single-center prospective comparative effectiveness randomized two-arm parallel-group superiority trial to test the effectiveness of linkage navigation and clinician referral among ED patients identified with untreated HCV with a primary hypothesis that linkage navigation plus clinician referral is superior to clinician referral alone when using treatment initiation as the primary outcome. Participants will be enrolled in the ED at Denver Health Medical Center (Denver, CO), an urban, safety-net hospital with approximately 75,000 annual adult ED visits. This trial was designed to enroll a maximum of 280 HCV RNA-positive participants with one planned interim analysis based on methods by O’Brien and Fleming. This trial will further inform the evaluation of cost effectiveness, disparities, and social determinants of health in linkage-to-care, treatment, and disease progression.

**Discussion:**

When complete, the DETECT Hep C Linkage-to-Care Trial will significantly inform how best to perform linkage-to-care among ED patients identified with HCV.

**Trial registration:**

ClinicalTrials.gov

ID: NCT04026867

Original date: July 1, 2019

URL: https://clinicaltrials.gov/ct2/show/NCT04026867

**Supplementary Information:**

The online version contains supplementary material available at 10.1186/s13063-022-07018-w.

## Introduction

Hepatitis C (HCV) is associated with significant morbidity and mortality in the USA and abroad [1–3]. The World Health Organization set a goal of eliminating viral hepatitis as a public health threat by 2030 and lists increasing HCV treatment as a key target of the elimination strategy [4]. Despite the high burden of disease caused by HCV and the heightened global awareness of the problem, many people living with HCV remain undiagnosed and therefore untreated [5]. Efforts to offer testing more broadly and more often are a crucial first step for increasing treatment. To that end, emergency departments (EDs) have been identified as key settings in which to offer HCV testing to individuals who might not otherwise interact with healthcare systems or be offered HCV testing [6–10].

**Fig. 1:**
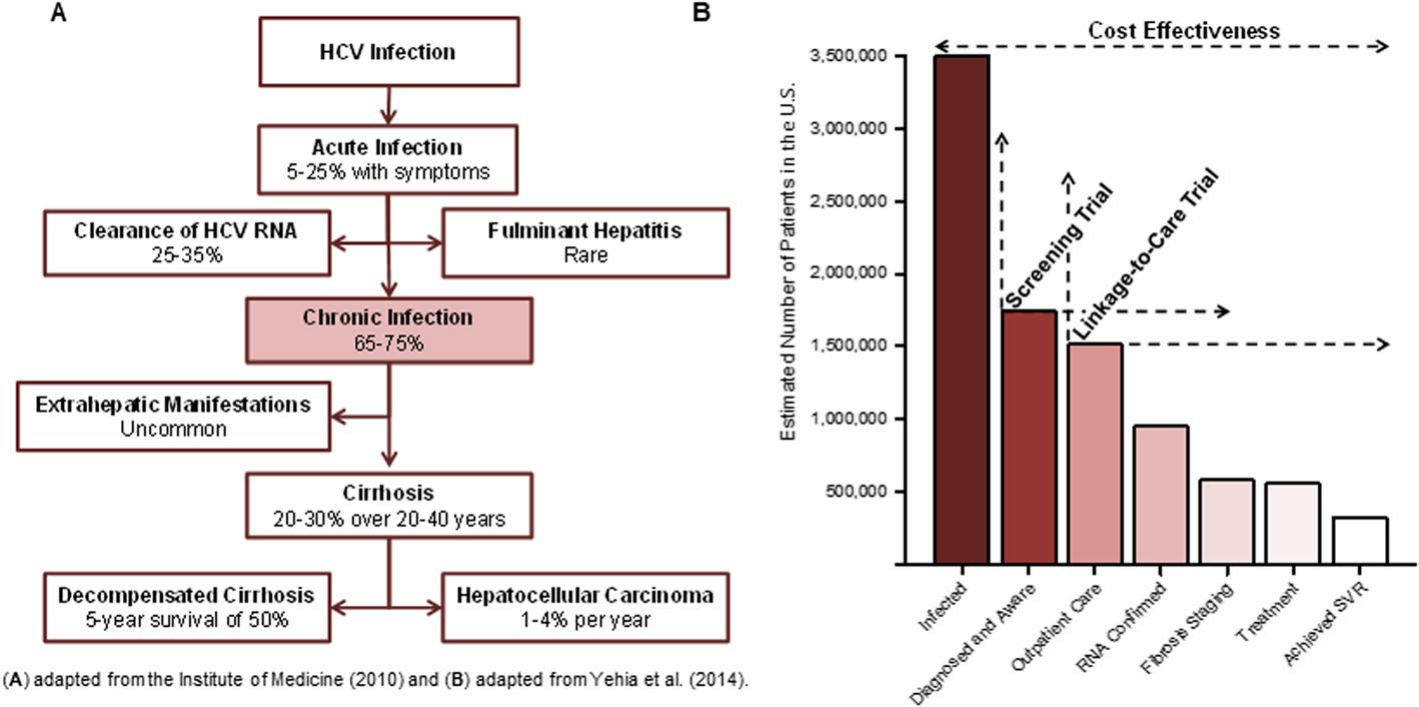
Natural progression of hepatitis C virus (HCV) infection (**A**) and the HCV care continuum (**B**). SVR, sustained virologic response 12 weeks of completion of treatment

Once diagnosed, linkage-to-care is the next critical component of the HCV care continuum (Fig. 1). Effective linkage-to-care has been a major challenge for ED-based HCV testing with studies reporting linkage for only 20–39% of those newly diagnosed [11, 12]. However, unlike HCV, many HIV testing programs have successfully implemented active linkage programs, yielding successful linkage rates of well over 80% within 3 months of HIV diagnosis [13]. Linkage navigators have been utilized in community-based HCV testing programs, but while the role of navigation has been described in these settings, the added benefit of a linkage navigator above and beyond standard clinician referral has not been evaluated in a prospective, comparative manner [14–16]. Additionally, the effectiveness of navigators for people who inject drugs (PWID), a traditionally under-treated population, has not been assessed. Given that HCV is now curable with short courses of well-tolerated, increasingly available medications, the critical elements to slowing the epidemic are now HCV testing and effective linkage to care [17–19]. In high-volume ED settings, a linkage navigator may improve laboratory follow-up results disclosure, posttest counseling, linkage-to-care, and ultimately treatment and cure.

The overall objectives of the *D*etermining *E*ffective *T*esting in *E*mergency Departments and *C*are Coordination on *T*reatment Outcomes (DETECT) for *Hep*atitis *C* (Hep C) study are to (1) compare the effectiveness of two forms of HCV screening—non-risk-based (nontargeted) and risk-based (targeted) HCV screening (Screening Trial)— [20] (2) compare the effectiveness of two forms of referral to treatment for ED patients identified with HCV—linkage navigation plus clinician referral versus clinician referral alone (Linkage-to-Care Trial), (3) measure and compare programmatic costs and project long-term clinical outcomes, costs, and cost effectiveness of ED-based HCV screening and linkage-to-care (Cost Evaluation), and (4) examine the effects of disparities and social determinants of health on linkage-to-care, treatment initiation, and treatment completion among patients identified with HCV in the ED (Disparities and Social Determinants of Health Evaluation).

This article describes the rationale and design for the Linkage-to-Care Trial and is reported in accordance with the SPIRIT Statement for clinical trials [21, 22]. The primary hypothesis for the Linkage-to-Care Trial is that linkage navigation in addition to clinician referral will significantly increase the proportion of newly HCV-diagnosed individuals who complete care visits and initiate treatment when compared to clinician referral alone. The protocol (Version 3.3, Date February 17, 2022) is provided in the Additional.

## Methods

The DETECT Hep C Linkage-to-Care Trial has been registered in ClinicalTrials.gov and will be reported in accordance with CONSORT guidelines [23].

### Trial design

**Fig. 2:**
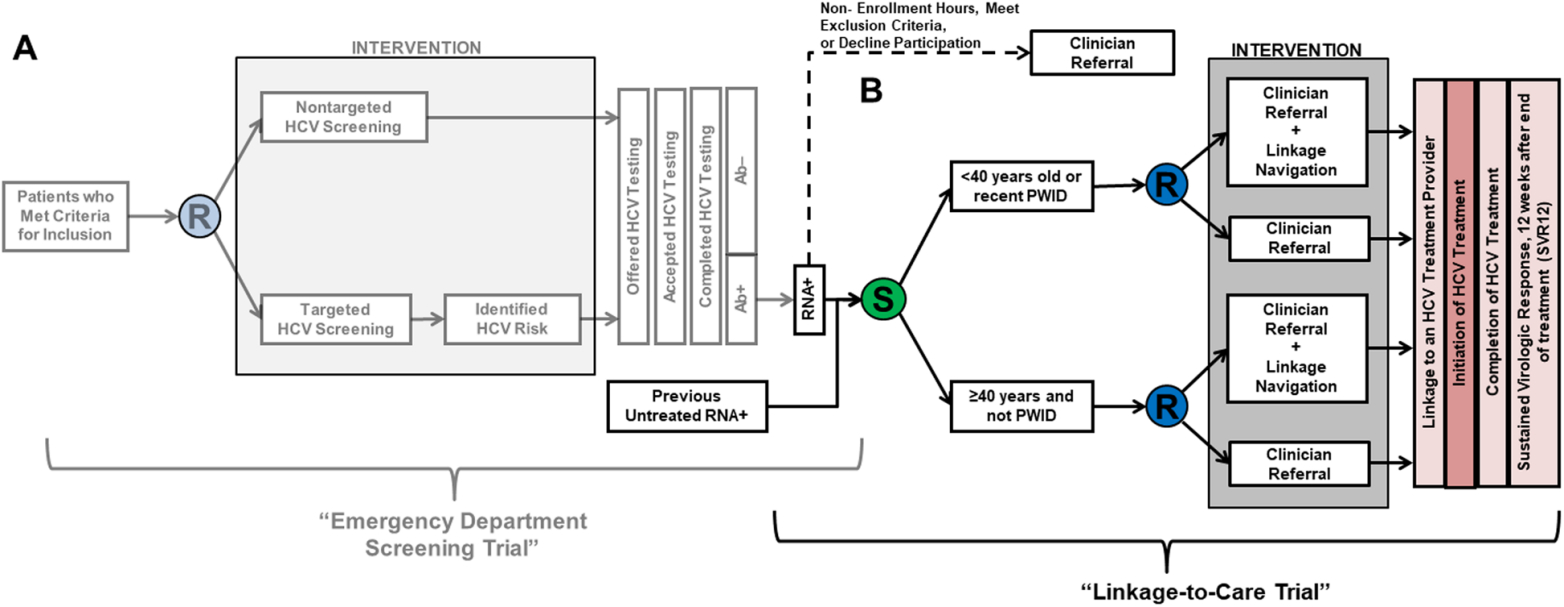
Study schematic for the The DETECT Hep C Trials, including the emergency department (ED) screening trial (**A**) and the ED linkage-to-care trial (**B**) [blue circle with “R” = randomization; green circle with “S” = survey/satisfaction; pink rectangle = primary outcome; light pink rectangle = secondary outcomes]. HCV, hepatitis C; PWID, person who injects drugs

For this trial, we will perform a single-center prospective randomized two-arm parallel-group superiority trial to evaluate the effectiveness of two linkage-to-care strategies for patients newly identified with untreated HCV in the ED from the DETECT Hep C Screening Trial or through an active surveillance process of previously diagnosed patients (Fig. 2) [20]. Permuted block randomization with two strata (i.e., those less likely to initiate treatment: <40 years of age or active injection drug use (IDU) [defined as IDU within 30 days of enrollment] or those more likely to initiate treatment: ≥ 40 years of age and without active IDU) and varying block sizes will be used to minimize imbalance and to ensure appropriate numbers of patients in subgroups.

### Study setting

This study will be performed at Denver Health (Denver, CO), a nationally recognized anchor institution, safety-net hospital, and integrated health care system that includes an acute care hospital and level I trauma center, 9 federally qualified community health centers, multiple specialty clinics including hepatology and infectious diseases, and the Public Health Institute at Denver Health.

### Eligibility criteria

Patients eligible for this trial will include those who meet criteria for inclusion in the DETECT Hep C Screening Trial and who test positive for HCV antibodies and those seen in the ED with previously diagnosed untreated active HCV confirmed in the electronic health record (EHR). Exclusions will include the following: prisoners and individuals who live outside of Colorado (given follow-up constraints), patients who speak a language other than English or Spanish (as the interview for participants will only be available in English and Spanish), and those who are pregnant (because HCV treatment is not currently approved for pregnancy). Those who are randomized with positive HCV antibody tests but have subsequent undetected HCV RNA results will be excluded from the study.

### Interventions

This trial will consist of a comparative evaluation of two forms of linkage-to-care among ED patients identified with untreated HCV. The first intervention, *clinician referral*, will serve as an “active control” and reference standard of care. All individuals included in this arm will be informed of their HCV antibody result by the ED treating provider who will be prompted to confirm that standardized language is included in discharge instructions as part of an automated after visit summary process, including post-testing information and specific follow-up guidance. Patients will also be instructed to access their electronic patient portal for their RNA test results or to call a designated results line. Patients with untreated active HCV (i.e., previously detected HCV RNA with no subsequent undetected RNA in their medical record) will receive tailored discharge instructions specific for individuals identified as having previously diagnosed but untreated HCV.

The second intervention, *clinician referral plus linkage navigation*, will consist of an additional service layered onto clinician referral and will incorporate protocols from Antiretroviral Treatment and Access Studies (ARTAS), the most influential studies of HIV linkage-to-care to date [13, 24]. Individuals allocated to this intervention will be contacted by a linkage navigator either during the ED visit (if during business hours) or the following business day (if during non-business hours). If the navigator does not meet with the patient at the time of enrollment, he or she will offer to meet with the patient in person or over the phone when convenient for the patient. For all individuals in this arm, a structured linkage navigation process will include (1) reiteration of posttest counseling messages and (2) assessment of the patient’s needs for medical insurance and substance use disorder treatment referral; navigation will also include (3) referral to an appropriate HCV treatment specialist with assistance scheduling appointments, (4) coordination of appointments with an enrollment specialist, if needed, (5) provision of social services (e.g., provision of transportation vouchers and referral to health insurance enrollment assistance, HIV care, local harm reduction services, and mental health services), (6) coordination of appointment scheduling including rescheduling missed appointments through the entire HCV treatment process, and (7) contacting patients after appointments to assess their understanding and any additional needs regarding engagement in HCV care (see “Linkage-to-Care Manual” in the Additional file 1).

Linkage navigators will undergo structured training prior to the start of the trial and will provide follow-up services for up to 18 months from the time of enrollment with a minimum of three attempted contacts for patients who are difficult to reach. For individuals who test negative for HCV RNA, linkage navigators will make no more than three attempts to contact the patient to (1) deliver and explain the RNA results, (2) discuss the risk of reinfection and future testing recommendations, and (3) provide resources for insurance enrollment and substance use disorder treatment, as needed.

### Allocation

#### Sequence generation, allocation concealment, and implementation

Participants who meet criteria for inclusion will be stratified into two groups (i.e., < 40 years of age or IDU or ≥ 40 years and no IDU) then randomly assigned using variable block randomization for each stratum to one of the two study arms. Sequence of random assignments will be generated in SAS Enterprise Guide Version 7.1 (SAS Institute, Inc., Cary, NC) and transferred into REDCap (Vanderbilt University, Nashville, TN) for use with enrollment. Integration of the randomization sequences into REDCap will allow for concealed allocation as research assistants will not be able to anticipate allocation. As such, patients who consent for participation will be randomly assigned to one of the two study arms by a research assistant.

### Blinding

Participants and ED staff will understand the conceptual goals of the trial but will be blinded to the details of the study hypotheses. Outcomes will be determined using research staff blinded to study group allocation and hypotheses.

### Recruitment

Recruitment of participants will occur by trained research staff during dedicated recruitment hours that will vary from 7 a.m. through 10 p.m., 7 days a week. The research team will work to optimize research staff coverage to correspond with ED census but will also create mechanisms to remotely screen and enroll. This includes enrollment over the phone for patients who are eligible for the trial but have already left the ED. These patients may also choose to return to the medical campus for in person enrollment. Participants will receive a $25 gift card for participation in the study.

### Participant timeline

Primary research activities, including screening for participation, consenting, and randomization by research assistants will typically occur during the patient’s ED visit, with an option for remote enrollment after the visit as described above. Clinician referral and follow-up guidance are standard procedure for all patients in the ED with newly or previously diagnosed HCV. For those allocated to the linkage navigation arm, a linkage navigator will meet with the patient during the ED visit if during standard business hours, or, if not during business hours (e.g., evenings, nights, weekends, or holidays), will make contact by telephone the next business day to facilitate an appointment for subsequent care with an HCV treatment specialist.

### Outcomes

The *primary outcome* for this trial will be initiation of HCV treatment within 12 months from the time of enrollment for those with a positive HCV RNA test. *Secondary outcomes* will include the following: (a) linkage to an HCV treatment specialist within 12 months of enrollment; (b) for individuals who report IDU, initiation or continuation of substance use disorder services within 12 months of enrollment; (c) completion of a full course of HCV treatment with direct-acting antiviral medicines (DAAs) within 18 months of enrollment; (d) sustained virologic response 12 weeks (SVR12) as measured by report of a negative HCV RNA test at least 12 weeks after completion of DAAs [17]; and (e) all outcomes within 18 months of enrollment. Individuals without evidence of an HCV-associated visit within 18 months from ED diagnosis or ED visit will be considered not linked to care. All outcome measures will be collected and verified via EHR review by trained research assistants blinded to study allocation.

### Sample size

**Fig. 3:**
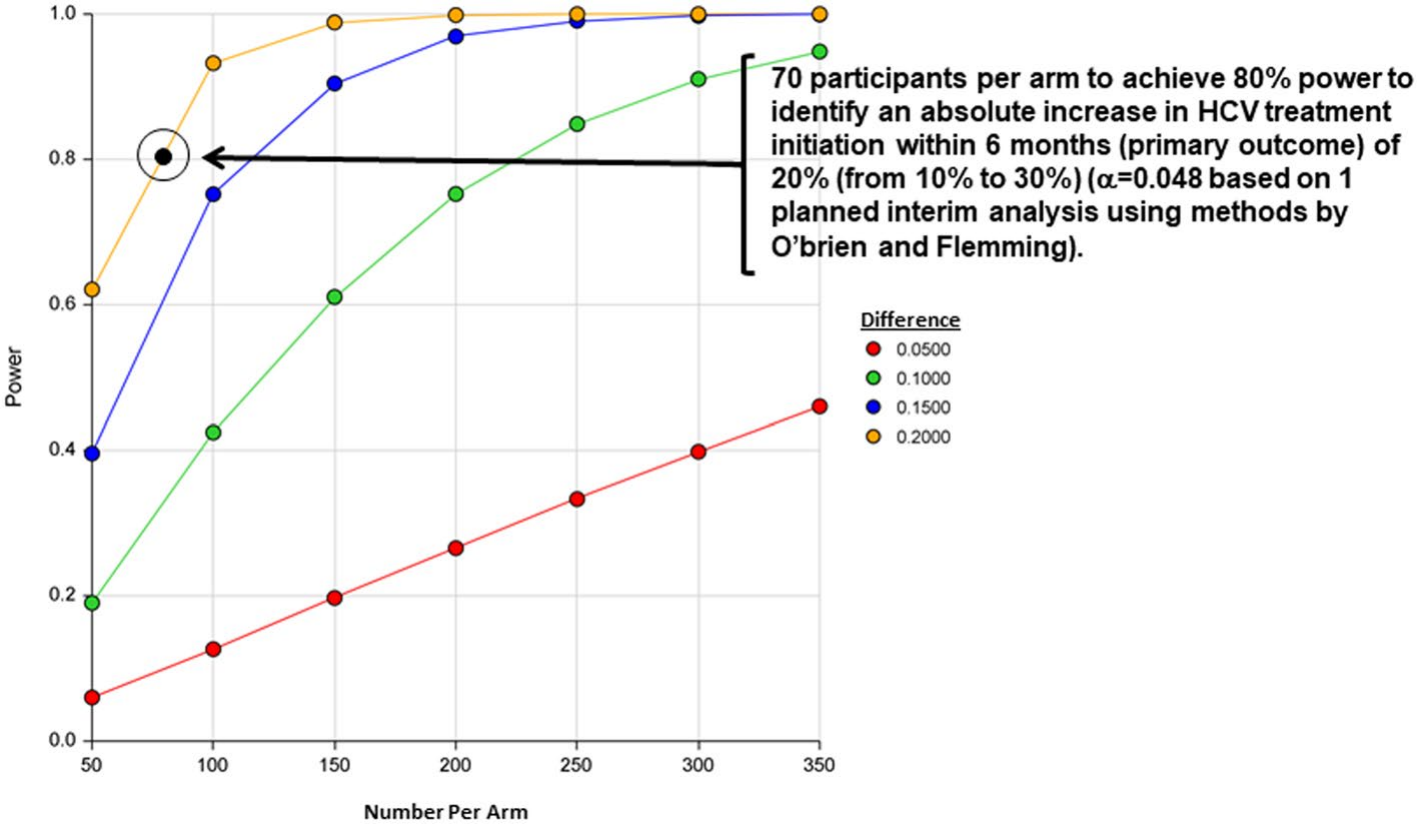
Sample size estimates for the DETECT Hep C Linkage-to-Care Trial

The *primary superiority hypothesis* for this trial was powered based on a conservative estimate of HCV treatment initiation of 10% for clinician referral and a hypothesized increase to 30% (absolute increase of 20%) for those allocated to the linkage navigation intervention (Fig. 3). This trial was powered based on the < 40 years of age or IDU stratum to ensure adequate power overall. As such, it was estimated that a minimum of 140 participants (70 per arm) will be required in each stratum for a total of 280 participants to achieve a power of 80%, using an alpha of 0.0492 based on methods for one interim analysis by O’Brien and Fleming. Using the approach by O’Brien-Fleming, the interim analysis will occur at the study’s halfway point, after 140 total patients (approximately 35 per arm) have been enrolled and outcomes data collected, and with an effectiveness threshold of *p* < 0.0054 for the primary outcome. A non-binding futility threshold of *p* > 0.5 will be used for the primary outcome. If the trial is not stopped after the interim analysis, it will proceed to enroll the full sample (280) with a significance effectiveness threshold of *p* < 0.0492.

### Data collection

During this trial, an assessment of barriers and facilitators to linkage to HCV care will be conducted through use of an audio computer administered self-interview (ACASI) offered to all enrolled patients after informed consent has been obtained but before randomization. ACASI has been shown to be a superior method for collecting potentially sensitive information in an efficient and anonymous format [25]. The survey will be offered in English and Spanish and will collect the following data for all enrolled patients: (1) ED visit information (unique study identifier, date/time of the visit), (2) demographics (age, gender, race, ethnicity, primary language), (3) payer information (commercial, Medicare, Medicaid, self), (4) detailed contact information, (5) details of randomization, (6) clinician referral information, (7) HCV RNA testing, (8) linkage navigation details, and (9) all outcome measures. Data from (1) through (6) will be collected prospectively by a trained research assistant during enrollment, data from (7) will be collected retrospectively by a trained research assistant, data from (8) will be collected prospectively by the linkage navigator, and data from (9) will be collected by a trained research assistant, blinded to intervention allocation and distinct from the research assistant who will perform enrollment. All retrospective data collection will use structured methods. In prior HIV studies, we have consistently obtained longitudinal follow-up for > 90% of HIV-diagnosed patients from the ED thus we anticipate collecting similarly high HCV follow-up data [26, 27].

### Data management

Data will be entered into a secure electronic database (REDCap, Vanderbilt University, TN) that will be developed to maximize valid data entry by including closed-response entries and range restrictions [28]. Data will then be transferred into native SAS format and cleaned prior to performing analyses using the most current version of SAS (SAS Institute, Inc., Cary, NC). The dataset will be locked, and all analyses will be performed by the study’s biostatistician in conjunction with the principal investigators while blinded to study allocation.

### Statistical methods

Analyses will be performed using the intention-to-treat principle. Bivariate statistical tests will be used to compare variables, including results from the ACASI surveys between study groups. Given the randomized design, the primary comparison will include the absolute percentage difference and risk ratio with 95% CIs for initiation of HCV treatment (primary outcome) and will be tested using chi-square or Fisher’s exact test. Kaplan-Meier and Cox proportional hazard regression will be used to estimate associations between the interventions and outcomes when modeled using time-to-event as an outcome. One interim effectiveness analysis is planned and will be performed by the study’s biostatistician while maintaining blinding to study arm allocation. Using the approach by O’Brien-Fleming, the interim analysis will occur at the study’s halfway point, after 140 total patients (approximately 35 per arm) have been enrolled and outcomes data collected, and with an effectiveness threshold of *p* < 0.0054 for the primary outcome. A non-binding futility threshold of *p* > 0.5 will be used for the primary outcome. If the trial is not stopped after the interim analysis, it will proceed to enroll the full sample (280) with a significance effectiveness threshold of *p* < 0.0492. The unit of analysis will be the patient. Secondary analyses will include comparisons of all other outcomes by study arm with subgroup analyses for the stratum of individuals < 40 years of age or PWID, age, gender, race, ethnicity, income, and education level.

Additional secondary analyses were planned a priori to evaluate disparities and the effects of social determinants of health (SDoH) on linkage, treatment initiation, and SVR12. Multivariable logistic regression analyses will be used to estimate the associations between gender and study outcomes while adjusting for confounders, including but not necessarily limited to age, race, ethnicity, housing status, HCV knowledge, and willingness to engage in care. Additionally, effect modifiers (e.g., race, ethnicity and gender, age and gender) will be evaluated. The statistical approach will be variable centered and will use hierarchical multivariable logistic regression to estimate associations between SDoH characteristics and linkage to care, treatment initiation, and SVR12. We will use a theoretical framework and prior studies to inform covariate selection to assess the relationships between SDoH and the outcomes.

Although we do not anticipate significant missing data, multiple imputation will be used in instances where variables have > 5% missingness. No adjustments will be made for loss to follow-up as patients will be considered not linked to care after 18 months from the time of entry into the study.

### Trial and data monitoring

In accordance with the National Institutes of Health (NIH) and the primary funding agency, the National Institute on Drug Abuse (NIDA), we have developed a Data and Safety Monitoring (DSM) plan, and the principal investigators and core, Denver-based, research team will maintain appropriate oversight and monitoring of the trial’s conduct in its entirety. As this trial is not a phase 3 trial and it was determined to be minimal risk to participants, no Data Safety Monitoring Board (DSMB) was required for this trial. The DSM plan was approved by the Colorado Multiple Institutional Review Board (COMIRB) and NIDA prior to initiating enrollment.

The core Linkage team, consisting of one of the study principal investigators, the Linkage-to-Care Trial project coordinator, and the linkage navigators, will meet quarterly to review cases, ensure fidelity to the linkage protocols, and troubleshoot challenging linkage situations. Data monitoring will be performed from the inception of participant enrollment through follow-up as captured in REDCap. The data manager will organize all data to understand total screening and enrollment, inclusion and exclusion criteria, consent, and study procedures associated with the interventions. Weekly study team meetings will occur to review screening and enrollment. Outcomes will be collected after each interim sample has been enrolled in an effort to complete interim analyses, performed by the study’s biostatistician.

### Special considerations during the COVID-19 pandemic

This trial has enrolled participants during the COVID-19 pandemic with enrollment suspended from March 13, 2020, through August 24, 2020, and from December 2, 2020, through January 29, 2021. Furthermore, during the COVID-19 pandemic, linkage-to-care and some healthcare visits transitioned to remote participant interactions. In an effort to incorporate the effect of delayed linkage-to-care and treatment initiation during the COVID-19 pandemic, we extended the time frame for which longitudinal outcomes will be collected from 12 months to up to 18 months, also modifying treatment initiation to within 18 months of enrollment.

### Research ethics approval, consent, and confidentiality

COMIRB serves as the institutional review board (IRB) of record and approved this trial on December 17, 2017. Participants will be enrolled in this trial after obtaining verbal consent. We requested, and have obtained, a waiver of documentation of written consent based on 45 CFR 46.117. We obtained a waiver of HIPAA authorization for participants in this study. Data collected as part of this project poses no more than a minimal risk of harm to privacy.

### Potential harms

Given the comparative effectiveness nature of this trial and that all research components will be fully integrated into standard care, the primary risks to patients included in this trial will be breach of confidentiality as all study procedures will be performed as routine medical care in the ED and as follow-up for those enrolled.

### Protocol amendments

Through May 16, 2022, 15 protocol amendments have occurred. See Protocol in the Additional file 1 for details.

### Access to data and dissemination policy

The primary results of this trial will be reported in accordance with CONSORT guidelines and published in a peer-reviewed journal.

## Discussion

When complete, the DETECT Hep C Linkage trial will represent the only randomized clinical trial to compare the effectiveness of different approaches to HCV linkage to care from the ED and to attempt to quantify the added benefit of a linkage navigator. Additionally, this trial will provide data on HCV linkage to care for patients identified with untreated HCV in an ED setting, a unique and promising venue for engaging patients with HCV. If linkage navigation plus clinician referral yields at least a 20% increase in HCV treatment initiation, this finding will have significant implications for clinical settings where HCV rates are high and where the addition of an HCV linkage navigator to hospital staff could yield major benefits to the community by decreasing HCV prevalence.

Rationale for the head-to-head comparison performed in this trial resulted from prior findings that clinician referral from ED settings, while current standard of care has limited effectiveness [9–11]. The benefits of a linkage navigator for HIV care after HIV diagnosis suggest that extension of linkage-to-care approaches for HCV are needed to facilitate treatment and, in turn, limit transmission [13, 24]. Until now, no prospective comparative evaluation of clinician referral to linkage navigation for HCV has been performed, particularly in an ED environment, and the findings will have important implications for clinical practice.

Potential limitations of this trial include its performance at a single academic medical center with experience performing infectious diseases screening in the ED and with integrated linkage-to-care services for HIV, but not HCV. While the navigation standard work was developed using an interdisciplinary group of programmatic and content experts and based on prior processes related to HIV screening and other navigation programs, success of navigation may be limited by complexities of navigating care for patients who are socially and economically vulnerable. Furthermore, secular trends related to the diagnosis and treatment of HCV may influence the numbers of patients identified with active chronic HCV, which may limit the ability to enroll, and conducting this trial during the COVID-19 pandemic has led to unanticipated delays and disruptions, including varying trends in access to care and the broader use of telehealth, which may also affect results. Finally, although the hypothesized effect was influenced by existing knowledge and content expertise, very little prior data existed to inform the hypothesized effect; therefore, depending on the true effect, this study may be underpowered.

## Trial status

Enrollment for this trial was initiated on November 20, 2019, with enrollment stoppage due to institutional policies related to the COVID-19 pandemic from March 13, 2020, through August 24, 2020, then again on December 2, 2020, through January 29, 2021, at which point enrollment resumed. The interim analysis occurred on August 1, 2022, without meeting a stopping rule. Complete enrollment is anticipated to finish in 2023.

## Supplementary Information

**Additional file 1.**

## Data Availability

The investigators will have access to the final trial dataset, and the principal investigators, Drs. Rowan and Haukoos, take responsibility for the trial as a whole.
